# Correlation analysis between foot deformity and diabetic foot with radiographic measurement

**DOI:** 10.3389/fcdhc.2023.1121128

**Published:** 2023-06-02

**Authors:** Xu Luo, Chun Zhang, Qiuhong Huang, Zhipeng Du, Xia Ni, Qinglian Zeng, Qingfeng Cheng

**Affiliations:** ^1^ Department of Endocrinology, The First Affiliated Hospital of Chongqing Medical University, Chongqing, China; ^2^ School of Public Health and Management, Chongqing Medical University, Chongqing, China

**Keywords:** diabetes mellitus, diabetic foot, hallux valgus, foot deformity, infection

## Abstract

**Background:**

Foot deformity is a risk factor for diabetic foot ulcer. This study was aimed to investigate the relationship between hallux valgus (HV) and diabetic foot through the radiographic measurement.

**Methods:**

The patients with diabetic foot hospitalizing in the Department of Endocrinology, the First Affiliated Hospital of Chongqing Medical University from September 2016 to June 2020 were selected. Then the foot plain X-ray radiographs were completed, and the size of HV angle (HVA) was measured. Their clinical data were collected, and the ulcer recurrence rate, amputation rate and mortality rate of the patients were followed up.

**Results:**

A total of 370 patients were included. According to HVA, patients were divided into non-HV group (HVA<15°), and mild (15°≤HVA ≤ 20°), moderate (20°<HVA ≤ 40°) and severe (HVA>40°) HV groups. The age, height, BMI, smoking history and glycosylated hemoglobin level among the non-HVA, mild, moderate, and severe HV group (P<0.05), while smoking history, HbA1c, eGFR and autonomic neuropathy were significantly lower in HV group than those in non-HV group (P<0.05). The ulcer area in patients with moderate HV was larger than that in non-HV patients, and the severity of infection in patients with severe HV was significantly higher than that the other three groups (P<0.05).

**Conclusion:**

The occurrence of HV is not only related to age and BMI, but also to the creatinine and eGFR level, autonomic neuropathy, lower limb arteriosclerosis occlusion, coronary heart disease and hypertension. Therefore, more attention should be paid to renal function screening, neuropathy screening and evaluation of lower extremity vascular lesions in patients with diabetes, especially those with moderate or higher HV.

## Highlights:

1. The occurrence of HV was related to demographic factors including age and BMI.
2. The occurrence of HV was related to the creatinine and eGFR level.
3. HV was related to autonomic neuropathy, lower limb arteriosclerosis occlusion, coronary heart disease and hypertension.
4. The severity of HV seems to be related to ulcer area and degree of infection.

## Introduction

Diabetes prevalence is increasing rapidly due to economic development and aging of population (1). Currently, China has become one of the most dramatic rises in diabetes prevalence in the world (1). Diabetes has changed from a rare disease to an epidemic in China. Diabetic foot is one of its serious complications. According to statistics, the prevalence of foot ulcer or gangrene in diabetic patients is as high as 6.3% (2) Diabetes-related foot complications have caused a huge economic and social burden (3, 4). The risk factors including diabetic neuropathy, peripheral vascular disease, limited joint mobility, foot deformity, increased plantar pressure, minor trauma, and a history of amputation and ulceration are associated with the development of the diabetic foot (5).

Diabetic neuropathy (DPN) is the most common chronic complication of diabetes and the most common predictor of foot ulcers (6). Among them, sensorimotor nerve damage is the most common complication of DPN, which can lead to decreased pain and temperature perception and loss of vibratory, tactile, and sensory perception (7). At the same time, the damage to the plantar skin and proprioception caused by peripheral neuropathy can cause abnormal movement and balance, resulting in gait instability and an increased risk of falls (8). Motor neuropathy could lead to structural changes of the foot due to Atrophy of intrinsic foot muscles and foot deformity (9, 10). Foot deformities and limited joint movement can lead to increased plantar pressure, excessive load carriage, and gait changes (11). Common foot deformities such as hallux valgus (HV) and hammer toe can cause bony protrusion, which can form ulcers on the back or top of the toe due to thin skin and subcutaneous tissue (12). In addition, patients with abnormal foot pressure tend to form corpus callosum at pressure points, then the following loss of protective sense and subtle repetitive damage could resulting in local tissue injury, inflammation, tissue necrosis, and finally ulcer (13, 14). On the other hand, diabetes can cause related vascular system damage, such as peripheral arterial disease, endothelial dysfunction (15), and blood flow regulation disorders caused by autonomic nervous system dysfunction (16). The effect of diabetic foot on the blood vessels can be manifested as muscle function impairment and loss of bone density, thereby affecting the biomechanics of the foot and the occurrence of foot ulcers (17–19). Foot deformity and biomechanical changes play an extremely critical role in the occurrence and development of various complications and concomitant diseases in the later stage of diabetes, and is the key factor in the occurrence of foot ulcers and amputations (20). Therefore, the evaluation of foot deformity is of great significance for further understanding the occurrence, development and prognosis of diabetic foot patients.

Studies have shown that digitized radiologic images are commonly used to assess the severity objectively in patients with HV deformity (21–24). The purpose of this study is aimed to better evaluate the importance of foot deformity in diabetic foot patients through the imaging measurement of HV deformity. The study could contribute to the early identification and intervention of diabetic foot to reduce plantar pressure and ulcer rate, and establish early warning signals of destructive complications.

## Patients and methods

### Study population and selection criteria

The patients with diabetic foot who were hospitalized in the Department of Endocrinology, the First Affiliated Hospital of Chongqing Medical University from September 2016 to June 2020 were selected, and the patients who met the inclusion criteria were selected as the research subjects.

The inclusion criteria of diabetic patients were: 1) who met the diagnostic criteria for type 2 diabetes (WHO 1999 criteria; 2) diagnosed with ischemic diabetic foot, neurological diabetic foot or mixed diabetic foot according to the Guidelines for the Prevention and Treatment of Diabetic Foot in China (2019 Edition) (II) and clinical examination; 3) with complete foot weight-bearing X-rays after admission.

The exclusion criteria were: 1) Patients with foot deformity, requiring dialysis treatment, diagnosed tumors, severe Malnourished patients, long-term bedridden due to cerebrovascular accident, or diagnosed with other neurological diseases were excluded.

This study was in line with the ethical principles of World Medical Association Declaration of Helsinki, and was approved by the ethics committee of the First Affiliated Hospital of Chongqing Medical University, and the guardians signed the informed consent.

### HVA measurement

**Figure 1:**
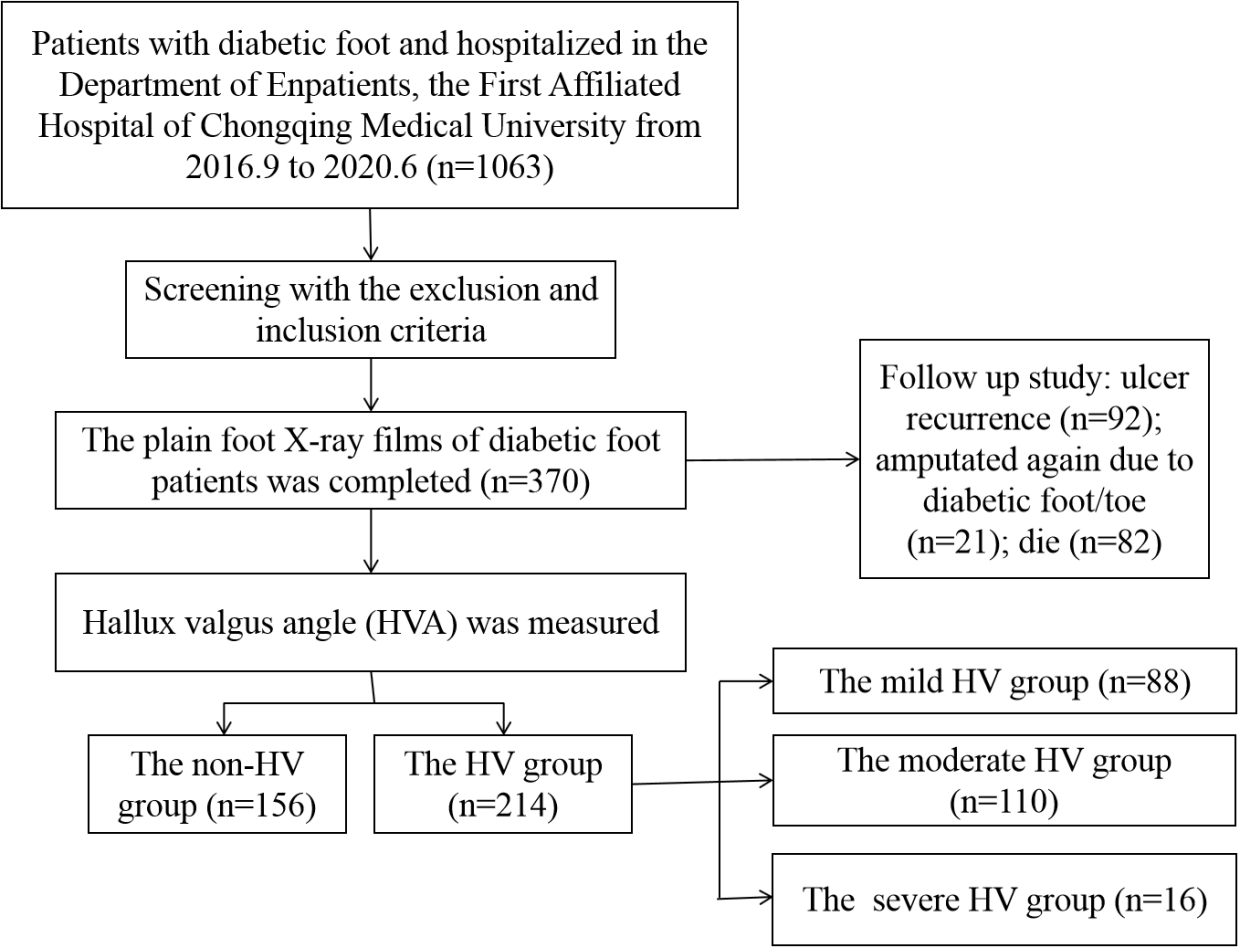
Flow-chart of study population and protocol.

After meeting the inclusion criteria, the plain foot X-ray films of diabetic foot patients were collected. The hallux valgus angle (HVA) was measured using Digimizer software (Version 4.6.2.0 copyright^©^2005-2014 MedCalc software) based on the foot plain X-ray radiographs of included patients **Figure 1**.

HVA was graded (22–24) as: 1) Normal: HVA<15°; 2) Mild: 15°≤HVA ≤ 20°; 3) Moderate: 20°<HVA ≤ 40°; 4) Severe; HVA>40°.

### General data of study population

The basic information including age, gender, height, weight, BMI, smoking/drinking history, blood pressure, and diabetes/diabetic foot disease course, underlying medical history including hypertension, dyslipidemia, coronary heart disease, and cerebrovascular disease, chronic complications of diabetes symptoms, and history of diabetic foot of patients were recorded. Clinical severity of infection was diagnosed with the perfusion, extent, depth, infection and sensation (PEDIS) classification system (25) and the Wagner’s grading system (25). The follow-up of included patients for recurrence, re-amputation/toe and mortality was completed in February 2022.

### Statistical analysis

The SPSS26.0 statistical software was used for statistical analysis, and K-S single-sample test was used to test whether data conformed to the normal distribution. Normally distributed data was expressed as mean ± standard deviation (X ± S), and analyzed by t test; non-normally distributed data was expressed as median (quartile), and analyzed by the rank sum test. Categorical and rank variables were expressed as percentages or frequencies, and comparisons between groups were performed using the chi-square test or Fisher’s exact test. Spearman correlation analysis was used to evaluate the relationship between HVA and related positive variables. Univariate logistic regression analysis was performed on all potential variables and the occurrence of HV. Then, all positive predictors (P<0.1) were analyzed by a multivariate logistic regression model. A P value < 0.05 was considered statistically significant. Graphs were drawn using GraphPad Prism 9.3.1.

## Results

### The HVA and the corresponding four groups

**Figure 2:**
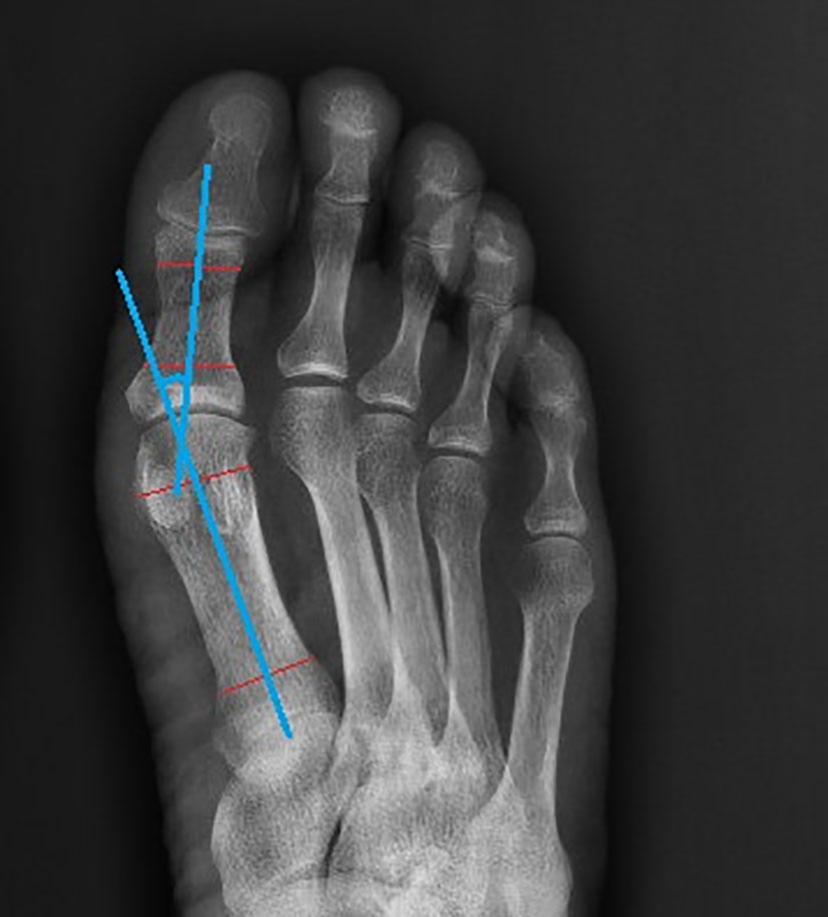
The measurement of hallux valgus angle.

**Figure 3:**
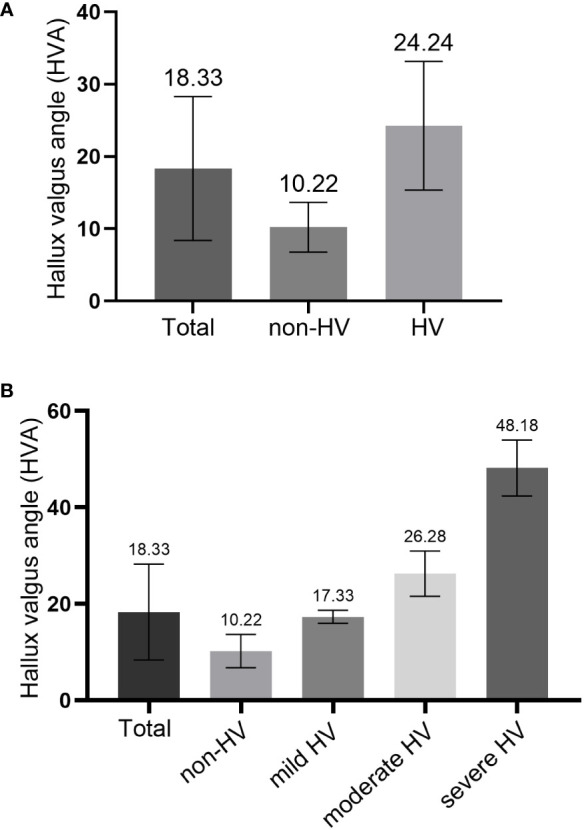
Comparison of hallux valgus angle (HVA) in different groups. **(A)** comparison between the non-HV group and HV group; **(B)** comparison among three HV groups.

A total of 370 patients with diabetic foot were included and the HVA was measured. **Figure 2** showed a flow chart describing the study population. The diseased side foot of patients with unilateral diabetic foot was measured, and the left foot of patients with bilateral diabetic feet were measured. The mean value of HVA was 18.33 ± 9.94°, the minimum value was 1.68°, and the maximum value was 58.37°. The enrolled participants were divided into groups according to HVA. There were 156 patients in the non-HV group and 214 in the HV group (**Figure 3A**). Moreover, there were 88 patients in the mild HV group, 110 patients in the moderate HV group, and 16 patients in the severe HV group (**Figure 3B**).

### Comparison between the HV group and non-HV group

The basic data of the total population was shown in **Table 1**. Age and BMI of the HV group were significantly higher than those of the non-HV group (P<0.05) (**Table 1**), and smoking history and the glycosylated hemoglobin level in the non-HV group were higher than those in the HV group (P<0.05). There were no significant differences in gender, residence, duration of diabetes, duration of diabetic foot disease, and drinking history between the two groups (P>0.05). In terms of diabetic complications and chronic complications, the proportion of autonomic neuropathy in the non-HV group (64.9%) was significantly higher than that in the HV group (38.8%). The incidence of coronary heart disease in the HV group was significantly higher than the non-HV group. Although there were no significant differences in the prevalence of retinopathy, neuropathy, diabetic nephropathy, hyperlipidemia and cerebrovascular disease between the two groups, the creatinine value of the HV group was significantly higher than that of the non-HV group (P<0.05), and the estimated glomerular filtration rate (eGFR) was significantly lower than that of the non-HV group (P<0.05). There were no significant differences in the history of foot ulcers, amputations, hospitalized amputation/toe rates, and the incidence of gangrene, necrotizing fasciitis, compartment syndrome, and Charcot foot between the two groups, nor the PEDIS classification (**Table 1**) and Wagner classification (data not shown).

**Table 1:** Comparison between the non-hallux valgus (HV) group and the hallux valgus group.

	Total (N=370)	Non-HV (N=156)	HV (N=214)	t/Z/χ^2^/H value	P value
Age (year)	64.66 ± 11.98	62.63 ± 11.00	66.61 ± 12.44	2.811	0.005
Gender (male/female)	251(69.5%)/113(30.5%)	116(74.4%)/40(25.6%)	141(65.9%)/73(34.1%)	3.052	0.081
Residence (Urban or rural)	263(71.1%)/107(28.9%)	115(73.7%)/41(26.3%)	148(69.2%)/66(30.8%)	0.912	0.339
BMI (kg/m2)	23.29 ± 3.29	22.68 ± 3.46	23.75 ± 3.07	2.952	0.003
Height(cm)	163.09 ± 8.00	163.85 ± 7.60	162.54 ± 8.29	1.483	0.139
Weight(kg)	62.04 ± 10.07	60.95 ± 10.47	62.87 ± 9.67	-1.729	0.085
Systolic pressure (mmHg)	134.17 ± 22.02	134.26 ± 21.08	135.13 ± 23.32	-0.368	0.713
Diastolic pressure (mmHg)	73.79 ± 12.99	74.60 ± 13.18	73.08 ± 12.99	1.104	0.27
Disease course of DM (year)	10.00(4.25,15.00)	10.00(5.00,17.00)	10.00(5.00,16.00)	0.977	0.329
Disease course of DF (month)	1.00(0.17,2.00)	1.00(0.13,3.00)	1.00(0.33,3.00)	0.680	0.496
Smoking history	192(51.9%)	91(58.3%)	101(47.2%)	4.483	0.034
Drinking history	139(37.6%)	65(41.7%)	74(34.6%)	1.932	0.165
HbA1c(%)	9.60(7.80,12.30)	9.50(6.80,12.60)	9.10(6.47,13.83)	3.504	<0.001
DPN	344(93.0%)	142(91.0%)	202(94.4%)	1.566	0.211
NSS stage					
Mild	26(15.1%)	11(14.5%)	15(15.6%)	2.713	0.258
Moderate	117(68.0%)	56(73.7%)	61(63.5%)		
Severe	29(16.9%)	76(11.8%)	20(20.8%)		
NDS stage					
Mild	22(11.6%)	9(10.8%)	13(12.1%)	0.088	0.957
Moderate	62(32.6%)	27(32.5%)	35(32.7%)		
Severe	106(55.8%)	47(56.6%)	59(55.1%)		
Autonomic neuropathy	43(50.0%)	24(64.9%)	19(38.8%)	5.740	0.017
Retinopathy	176(47.6%)	72(46.2%)	104(48.6%)	0.216	0.642
DKD	190(51.4%)	75(48.1%)	115(53.7%)	1.158	0.282
eGFR (ml/min/1.73m²)	84.80(57.87,107.55)	86.18(61.45,108.18)	73.10(49.90,99.30)	-2.935	0.003
Creatinine(μmol/L)	76.50(57.00,107.50)	72.50(55.75,102.00)	80.00(62.50,118.50)	-2.7	0.007
Arteriosclerosis obliterans	102(27.9%)	34(22.1%)	102(32.1%)	4.435	0.035
Hypertension	199(53.8%)	71(45.5%)	128(59.8%)	7.423	0.006
Hyperlipidaemia	91(25.6%)	42(28.4%)	49(23.6%)	1.056	0.304
Coronary heart disease	79(21.4%)	22(14.1%)	57(26.6%)	8.440	0.004
Cerebrovascular Disease	42(11.4%)	17(10.9%)	25(11.7%)	0.055	0.814
History of foot ulcers	104(28.1%)	44(28.2%)	60(28.0%)	0.001	0.972
History of amputation/Apodization	30(8.1%)	11(7.1%)	19(8.9%)	0.404	0.525
The hospital amputation/Apodization	67(18.1%)	31(19.9%)	36(16.8%)	0.566	0.452
P (vessel)					
1	224(60.9%)	100(64.5%)	124(58.2%)	1.723	0.423
2	67(18.2%)	27(17.4%)	40(18.8%)		
3	77(20.9%)	28(18.1%)	49(23.0%)		
E (area)	4.00(1.59,12.00)	4.00(1.50,9.00)	4.00(2.00,13.50)	-1.837	0.066
D (deepness)					
1	20(5.4%)	7(4.5%)	13(6.1%)	0.896	0.891
2	169(45.7%)	70(44.9%)	99(46.3%)		
3	179(48.4%)	78(50.0%)	101(47.2%)		
I (infection)					
1	10(2.7%)	2(1.3%)	8(3.7%)	2.482	0.479
2	124(33.5%)	53(34.0%)	71(33.2%)		
3	214(57.8%)	93(59.6%)	121(56.5%)		
4	22(5.9%)	8(5.1%)	14(6.5%)		
S (sensation)	329(88.9%)	137(87.8%)	192(89.7%)	0.33	0.566
ABI	1.10(0.90,1.18)	1.09(0.98,1.18)	1.11(0.85,1.17)	-0.57	0.568
Thanatosis	66(17.8%)	23(14.7%)	43(20.1%)	1.762	0.184
Necrotizing fasciitis	39(10.5%)	15(9.6%)	24(11.2%)	0.245	0.621
Compartment syndrome	12(3.2%)	6(3.8%)	6(2.8%)	0.312	0.576
Charcot’s foot	19(5.1%)	10(6.4%)	9(4.2%)	0.9	0.343

BMI, body mass index; DM, diabetes mellitus; DF, diabetic foot; HbA1c, Hemoglobin A1c; DPN, diabetic neuropathy; NSS, neuropathy symptom score; NDS, neuropathy disability score; DKD, diabetic kidney disease; eGFR, estimated glomerular filtration rate; ABI, ankle brachial index.

### Comparison among the non-HV, mild, moderate, and severe HV group

**Table 2** showed that there were significant differences in age, height, BMI, smoking history and glycosylated hemoglobin level among the non-HV, mild, moderate, and severe HV group (P<0.05). The BMI of the mild, moderate and severe HV groups were significantly higher than those of the non-HV group, but there was no statistical difference between the three groups; the height of the severe HV group was significantly lower than the other three groups (all P < 0.05). The number of patients with smoking history in the non-HV group was significantly higher than that in the moderate and severe HV group, and the smoking history of the patients in the severe HV group was also significantly higher than that in the moderate HV group. The level of glycosylated hemoglobin in the non-HV group was significantly higher than that in the mild, moderate and severe HV groups (P<0.05).

**Table 2:** Comparison among the non-hallux valgus (HV), mild, moderate, and severe HV group.

	Total (N=370)	non-HV (N=156)	mild HV (N=88)	moderate HV (N=110)	severe HV (N=16)	Z/χ2/H value	P value
Age(year)	64.66 ± 11.98	62.63 ± 11.00	65.56 ± 11.77	66.44 ± 12.90^a^	68.31 ± 13.25	8.724	0.033
Gender (male/female)	257(69.5%)/113(30.5%)	116(74.4%)/40(25.6%)	60(68.2%)/28(31.8%)	74(67.3%)/36(32.7%)	7(43.8%)/9(56.3%)	7.066	0.069
Residence (Urban or rural)	263(71.1%)/107(28.9%)	115(73.7%)/41(26.3%)	55(62.5%)/33(37.5%)	81(73.6%)/29(26.4%)	12(75.0%)/4(25.0%)	4.149	0.246
BMI (kg/m2)	23.29 ± 3.29	22.68 ± 3.46	23.73 ± 3.09^a^	23.60 ± 3.16^a^	24.80 ± 2.38^a^	11.274	0.010
Height(cm)	163.09 ± 8.00	163.85 ± 7.60	162.90 ± 8.23	163.02 ± 8.36	157.53 ± 6.81^a,b,c^	7.996	0.046
Weight(kg)	62.04 ± 10.07	60.95 ± 10.47	63.34 ± 9.58	62.60 ± 9.98	62.20 ± 8.59	1.093	0.352
Systolic pressure (mmHg)	134.17 ± 22.02	134.26 ± 21.08	136.11 ± 24.20	134.25 ± 23.20	135.88 ± 20.20	0.164	0.921
Diastolic pressure (mmHg)	73.79 ± 12.99	74.60 ± 13.18	72.71 ± 13.06	72.22 ± 12.72	80.94 ± 12.68	2.546	0.056
Disease course of DM (year)	10.00(4.25,15.00)	10.00(4.25,15.00)	10.00(5.00,18.00)	10.00(7.00,17.00)	8.00(3.50,15.00)	3.534	0.316
Disease course of DF (month)	1.00(0.17,2.00)	1.00(0.33,2.00)	0.90(0.28,2.00)	1.00(0.33,3.50)	1.00(1.00,18.75)	6.257	0.100
Smoking history	192(51.9%)	91(58.3%)	48(54.5%)	49(44.5%)^a^	4(25.0%)^a,b^	9.827	0.020
Drinking history	139(37.6%)	65(41.7%)	36(40.9%)	32(29.1%)	6(37.5%)	4.906	0.179
HbA1c(%)	9.60(7.80,12.30)	10.50(8.30,12.60)	9.10(7.58,11.08)^a^	9.30(7.30,11.25)^a^	9.05(6.65,10.25)^a^	13.521	0.004
DPN	342(91.0%)	142(95.5%)	84(94.4%)	102(87.5%)	14(92.9%)	2.812	0.422
NSS stage							
Mild	26(15.1%)	11(14.5%)	7(16.7%)	5(10.9%)	3(37.5%)	8.327	0.215
Moderate	117(68.0%)	56(73.7%)	26(61.9%)	30(65.2%)	5(62.5%)		
Severe	29(16.9%)	9(11.8%)	9(21.4%)	11(23.9%)	0(0.00%)		
NDS stage							
Mild	22(11.6%)	9(10.8%)	5(10.9%)	6(11.3%)	2(25.0%)	3.587	0.732
Moderate	62(32.6%)	27(32.5%)	15(32.6%)	16(30.2%)	4(50.0%)		
Severe	106(55.8%)	47(56.6%)	26(56.5%)	31(58.5%)	2(25.0%)		
Autonomic neuropathy	43(50.0%)	24(64.9%)	7(46.7%)	9(32.1%)	3(50.0%)	6.952	0.073
Retinopathy	175(47.7%)	72(46.5%)	50(57.5%)	47(43.1%)	6(37.5%)	5.011	0.172
DKD	190(51.4%)	75(48.4%)	42(47.7%)	63(57.8%)	10(62.5%)	3.607	0.313
eGFR (ml/min/1.73m²)	80.27(56.35,101.45)	86.18(61.45,108.18)	74.15(52.50,98.13)^a^	72.85(46.47,99.98)^a^	71.30(59.32,86.98)	8.869	0.031
Creatinine(μmol/L)	78.00(60.00,109.00)	72.50(55.75,102.00)	80.00(60.00,112.00)	80.50(64.50,126.00)	82.00(62.25,118.00)	7.565	0.056
Arteriosclerosis obliterans	102(27.9%)	34(22.1%)	27(31.0%)	38(34.5%)	3(20.0%)	5.904	0.116
Hypertension	199(53.8%)	71(45.5%)	54(61.4%)^a^	63(57.3%)	11(68.8%)	8.308	0.040
Hyperlipidaemia	91(25.6%)	42(28.4%)	19(22.6%)	24(22.2%)	6(37.5%)	2.831	0.418
Coronary heart disease	79(21.4%)	22(14.1%)	26(29.5%)^a^	28(25.5%)^a^	3(18.8%)	9.541	0.023
Cerebrovascular Disease	42(11.4%)	17(11.0%)	10(11.4%)	14(12.7%)	1(6.3%)	0.642	0.887
History of foot ulcers	104(28.1%)	44(28.4%)	22(25.0%)	33(30.3%)	5(31.3%)	0.751	0.861
History of amputation/Apodization	30(8.1%)	11(7.1%)	5(5.7%)	10(9.1%)	4(25.0%)	7.199	0.066
The hospital amputation/Apodization	67(18.1%)	31(19.9%)	18(20.5%)	18(16.4%)	0(0.0%)	4.418	0.22
Thanatosis	66(17.8%)	23(14.7%)	21(23.9%)	22(20.2%)	0(0.0%)	7.067	0.068
P (vessel)							
1	224(60.9%)	100(64.5%)	51(58.6%)	61(55.5%)	12(75.0%)	4.805	0.569
2	67(18.2%)	27(17.4%)	18(20.7%)	20(18.2%)	2(12.5%)		
3	77(20.9%)	28(18.1%)	18(20.7%)	29(26.4%)	2(12.5%)		
E(area)	4.00(1.59,12.00)	4.00(1.50,9.00)	4.50(2.00,15.00)	6.00(2.00,15.75)^a^	2.25(1.00,6.13)^c^	9.605	0.022
D (deepness)							
1	20(5.4%)	7(4.5%)	5(5.7%)	8(7.3%)	0(0.0%)	10.858	0.286
2	169(45.7%)	70(44.9%)	41(46.6%)	45(40.9%)	13(81.3%)		
3	179(48.4%)	78(50.0%)	42(47.7%)	56(50.9%)	3(18.8%)		
I (infection)					a,b,c	10.495	0.015
1	10(2.7%)	2(1.3%)	1(1.1%)	7(6.4%)	0(0.0%)		
2	124(33.5%)	56(35.9%)	34(38.6%)	32(29.1%)	2(12.5%)		
3	214(57.8%)	90(57.8%)	46(52.3%)	65(59.1%)	13(81.3%)		
4	22(5.9%)	8(5.1%)	7(8.0%)	6(5.5%)	1(6.3%)		
S (sensation)	329(88.9%)	137(87.8%)	78(88.6%)	99(90.0%)	15(93.8%)	0.708	0.871
ABI	1.10(0.90,1.18)	1.09(0.98,1.18)	1.11(0.89,1.17)	1.08(0.72,1.17)	1.13(1.05,1.18)	2.086	0.555
Necrotizing fasciitis	39(10.5%)	15(9.6%)	14(15.9%)	10(9.1%)	0(0.0%)	4.692	0.171
Compartment syndrome	12(3.2%)	6(3.8%)	4(4.5%)	2(1.8%)	0(0.0%)	1.407	0.675
Charcot’s foot	19(5.1%)	10(6.4%)	1(1.1%)	5(4.5%)	3(18.8%)^a,b.c^	9.550	0.023

BMI, body mass index; DM, diabetes mellitus; DF, diabetic foot; HbA1c, Hemoglobin A1c; DPN, diabetic neuropathy; NSS, neuropathy symptom score; NDS, neuropathy disability score; DKD, diabetic kidney disease; eGFR, estimated glomerular filtration rate; ABI, ankle brachial index. A. Compared with non-bunion group, p<0.05; b. Compared with mild HV group, p<0.05; c. Compared with moderate HV group, p<0.05.

The incidence of hypertension in the mild HV group was significantly higher than that in the non-HV group, and the incidence of coronary heart disease in the mild HV and moderate group was higher than that in the non-HV group (P<0.05). The eGFR level of the mild and severe HV groups were significantly lower than that of the non-HV group.

The proportion of Charcot’s foot of the severe HV group was significantly higher than the other three groups. The comparison of PEDIS classification among the four groups showed that the ulcer area in patients with moderate HV was greater than that in patients without HV (**Table 2**); while the severity of infection in patients with severe-HVA was greater than that in the other three groups (P<0.05). However, no statistically significant difference was found in the Wagner grading comparison between the 4 groups (data not shown).

### The follow-up analysis

A total of 370 patients were followed up by telephone, of which 282 patients or their family members answered relevant follow-up questions, and 88 patients were lost to follow-up due to wrong phone calls, empty numbers, shutdown, shutdown, and refusal to answer. The median follow-up time of the 282 patients was 3.92 (3.00, 5.08) years, of which the median follow-up time of non-HV patients (N=110) was 3.96 (3.00, 5.00) years, and the median follow-up time of HV patients (N=127) was 3.96 (3.00, 5.00) years. The follow-up time was 3.92 (3.02, 5.23) years, and there was no statistical difference between the two groups (Z=0.258, P=0.796). Of the 282 patients, 92 patients had ulcer recurrence (32.6%), 21 patients were amputated again due to diabetic foot/toe (7.4%), and 82 patients died due to diabetic foot, diabetes-related complications, or cardiovascular and cerebrovascular events (29.1%). However, there was no significant difference in recurrence, re-amputation/toe and mortality between the non-HV group and the HV group (P>0.05).

### Correlation analysis and logistic regression analysis

Spearman correlation analysis showed that the angle of HV was positively correlated with age (spearman correlation coefficient r = 0.138; p=0.008), BMI (r = 0.149; p=0.006), and arteriosclerosis obliterans (r = 0.106; p=0.043), hypertension (r = 0.116; p=0.026) and coronary heart disease (r = 0.11; p=0.035), while it was negatively correlated with smoking history (r = -0.174; p= 0.001), HbA1c (r = -0.17; p=0.001), autonomic neuropathy (r = -0.278; p=0.007), and eGER (r = -0.142; p=0.014).

After univariate and multivariate logistic regression analysis, autonomic neuropathy was found to be a protective factor for the development of HV (**Table 3**).

**Table 3:** The results of multivariate logistic regression analysis on hallux valgus.

Vatiate	Modle 1			Modle 2			Modle 3		
	P value	OR	95% CI	P值	OR	95% CI	P值	OR	95% CI
Age(year)	0.013	1.026	1.005-1.047				0.974	0.999	0.948-1.054
BMI(kg/m^2^)	0.015	1.092	1.017-1.172				0.953	0.995	0.852-1.163
Drinking history	0.044	0.622	0.392-0.987				0.062	0.377	0.136-1.05
HbA1c(%)	0.011	0.895	0.821-0.975				0.051	0.817	0.669-1.010
Autonomic neuropathy	0.22222			0.006	0.348	0.164-737	0.049	0.374	0.14-0.997
Arteriosclerosis obliterans				0.472	1.444	0.53-3.931	0.218	2.443	0.589-10.129
Hypertension				0.026	2.417	1.112-5.256	0.368	1.625	0.564-4.682
Coronary heart disease				0.563	1.595	0.328-7.752	0.298	2.606	0.429-15.819

Modle 1: Age, BMI, smoking history and HbA1c were independent variables, and bunion was taken as dependent variables;

Modle 2: Autonomic neuropathy, arteriosclerosis occlusion, hypertension and coronary heart disease were independent variables, and bunion valgus was used as dependent variables;

Modle 3: Age, BMI, smoking history and HbA1c were adjusted on the basis of model 2.

## Discussion

HV as one of the common foot deformities (26) has a morbidity rate of only 2-4% (27) in the general population, and top to 57.8% in patients with diabetic foot. Nix et al. indicated that HV is correlated with age and BMI (28), this is consistent with the our study. In addition, this study found that HV was positively related to arteriosclerosis obliterans, hypertension, coronary heart disease, and was negatively correlated with smoking history, HbA1c, autonomic neuropathy and eGER. The results also indicated that autonomic neuropathy could be the protective factor for the development of HV.

Among the 370 patients, there was no difference between the occurrence of HV and diabetic nephropathy, but the patients with HV had higher creatinine and lower eGFR, suggesting that the renal function of HV patients was worse than that of non-HV patients. The incidence of lower limb arteriosclerosis obliterans, hypertension and coronary heart disease in patients with HV was significantly higher than that in patients without HV. Diabetes can cause peripheral arterial disease, endothelial dysfunction and autonomic nervous system dysfunction, etc., resulting in blood flow regulation disorders (16), which in turn affects the blood vessels of the feet, manifested as muscle function damage and loss of bone density, thus affecting the foot. Changes in foot biomechanics (17–19), which may be the cause of foot deformities related to vascular lesions.

HV has been identified as a risk factor for foot ulcers (5). By grading the severity of HV according to the PEDIS classification system, it was found that the ulcer area of moderate HV patients was significantly larger than that of non-HV patients, and the infection severity of severe HV patients was significantly higher than that of non-HV patients, mild and moderate HV patients. Foot deformity increases the peak plantar pressure (26), further increasing the pressure load of the plantar (29, 30), leading to subcutaneous bleeding and resulting in foot ulcers (26). Moreover, patients with severe HV had significantly higher percentage of Charcot’s foot than the other groups, this suggesting that severe HV may contribute to other foot deformities. While, after follow-up study, there was no difference in recurrence rate, re-amputation/toe rate and mortality rate between the HV group and the non-HV group. Therefore, the influence of HV on the severity of diabetic foot and related complications needs to be further studied. In addition, the present study found that HV was not associated with the course of diabetes, but fewer smokers and lower HbA1c level were found in HV patients than that in non-HV patients, it possibly because the latter were older, tended to be more severe, and received an enhanced glycemic regimen.

Moreover, the increased plantar pressure and load in diabetic patients with HV may be a result of their larger ulcer size, while the increased ulcer size also increases the risk of infection. Studies suggest that HV is related to the degree of infection (31), but current studies have not further broken down acute infection, chronic infection, etc. Therefore, further exploration is needed for the specific type of infection and the type of microorganism infected, combined with variables such as ulcer site, depth of infection, and ulcer area in the follow-up in future studies.

At present, there only a few articles have reported that neuropathy is related to the occurrence of foot deformity (32), and neuropathy is not an important risk factor of HV (33). Studies have shown that motor neuropathy can cause muscle weakness and lead to deformity (34) and the reduction of the ankle dorsiflexion range is related to the severity of metatarsophalangeal joint deformity (32). Therefore, the relationship between muscle weakness and joint motion limitation and foot deformity in diabetic foot patients could be further investigated, and further research is needed to clarify whether there is a potential relationship between autonomic neuropathy and foot deformity.

However, there are some disadvantages of this study that must be considered: this study was a cross-sectional study; the included patients were all diagnosed with diabetic foot, and there was no diabetes without diabetic foot as control; the objects were all from the same hospital with severe condition, therefore there could be selection bias.

In conclusion, the occurrence of HV in diabetic foot was not only related to age and BMI, but also related to the occurrence of creatinine level, HbA1c level, eGFR level, autonomic neuropathy, lower limb arteriosclerosis occlusion, coronary heart disease and hypertension. Moreover, HV seems to be related to ulcer area and severity of infection, and autonomic neuropathy was found to be a protective factor for the development of HV. Therefore, patients with diabetes mellitus, especially those with moderate or higher HV, should pay more attention to their renal function, neuropathy screening and assessment of lower extremity vascular lesions, such as ABI, TBI, lower extremity vascular ultrasound, and even CTA of lower extremity arteries if necessary to assess the vascular lesions more visually. At the same time, in addition to strengthening the screening for complications, it is also important to assess the ulcer area and the degree of infection. When the infection is suspected to have invaded the bone, enhanced MRI or probe-to-bone test should be performed to assess whether it is combined with osteomyelitis.

## Data availability statement

The original contributions presented in the study are included in the article/supplementary material. Further inquiries can be directed to the corresponding authors.

## Ethics statement

The studies involving human participants were reviewed and approved by First Affiliated Hospital of Chongqing Medical University. The patients/participants provided their written informed consent to participate in this study. Written informed consent was obtained from the individual(s) for the publication of any potentially identifiable images or data included in this article.

## Author contributions

Conception and design of the research: XL and CZ. Acquisition of data: QH and ZD. Analysis and interpretation of data: XN, QZ and QC. Drafting the manuscript: XL and CZ. Revision of manuscript for important intellectual content: QZ and QC. All authors contributed to the article and approved the submitted version.
