# Erratum: Correlation analysis between foot deformity and diabetic foot with radiographic measurement

**DOI:** 10.3389/fcdhc.2024.1392508

**Published:** 2024-03-06

**Authors:** Frontiers Production Office

**Affiliations:** Frontiers Media SA, Lausanne, Switzerland

**Keywords:** diabetes mellitus, diabetic foot, hallux valgus, foot deformity, infection

Due to a production error, an incorrect name was provided for “the First Affiliated Hospital of Chongqing Medical University” in the Patients and methods section.

A correction has been made to the section Patients and methods, subsection Study population and selection criteria.

“The patients with diabetic foot who were hospitalized in the Department of Endocrinology, the First Affiliated Hospital of Chongqing Medical University from September 2016 to June 2020 were selected, and the patients who met the inclusion criteria were selected as the research subjects.

[ … ]

This study was in line with the ethical principles of World Medical Association Declaration of Helsinki, and was approved by the ethics committee of the First Affiliated Hospital of Chongqing Medical University, and the guardians signed the informed consent.”

The publisher apologizes for this mistake. The original version of this article has been updated.

